# Solid State NMR for Mechanistic Exploration of CO_2_ Adsorption on Amine-Based Silica Adsorbents

**DOI:** 10.1021/acsomega.4c11221

**Published:** 2025-02-20

**Authors:** Mohammed Jasil, Brijith Thomas

**Affiliations:** † Science Division, New York University Abu Dhabi, P.O. Box 129188, Abu Dhabi 129188, United Arab Emirates; ‡ Mubadala Arabian Center for Climate and Environmental Sciences, New York University Abu Dhabi, P.O. Box 129188, Abu Dhabi129188, United Arab Emirates

## Abstract

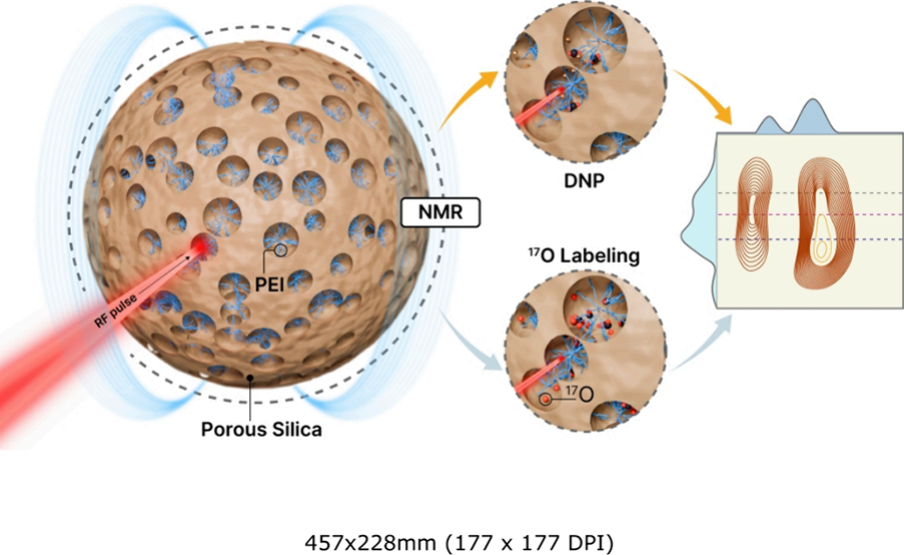

Mitigating atmospheric carbon dioxide concentrations is crucial because elevated CO_2_ levels drive climate change by enhancing the greenhouse effect, leading to global warming, extreme weather events, ocean acidification, loss of biodiversity, and significant socioeconomic and health challenges for ecosystems and human populations. The necessity to reduce atmospheric carbon dioxide levels has led to the creation of novel materials designed to effectively capture and convert CO_2_ using carbon capture and utilization methods. A diverse array of materials such as metal–organic frameworks, covalent organic frameworks, porous carbon, zeolites, and amine functionalized silica has been reported for efficient carbon dioxide capture. Notably, amine-functionalized silica has emerged as one of the most extensively studied materials in the field of carbon dioxide capture. Solid-state NMR is a powerful spectroscopic technique for analyzing amine-based silica adsorbents, as it provides detailed, nondestructive molecular insights into structure, interactions, and adsorption mechanisms that are challenging to resolve using traditional techniques like infrared spectroscopy and BET (Brunauer-Emmett-Teller). Solid-state NMR, particularly magic angle spinning (MAS) NMR, demonstrates significant potential in providing high-resolution insights into atomic-level interactions and dynamics. This minireview will explore how solid-state NMR spectroscopy and its advancements are effective in investigating the amine immobilization and stabilization mechanism on silica, probing the local structures of CO_2_ adsorption species, and assessing the influence of varying conditions on the performance of adsorbents. The information obtained through the application of various solid-state NMR experiments is emphasized, along with strategies for further enhancing this knowledge.

## 1 Introduction

Recent advancements in the research on carbon dioxide (CO_2_) capture and utilization have gained significant momentum, as this area of study offers potential solutions to two critical challenges: global energy demand and the environmental pollution resulting from CO_2_ emissions.^1^ The capture of carbon dioxide emitted from specific sources, such as automobile emissions, through direct air capture (DAC) necessitates the development of advanced materials specifically designed for efficient CO_2_ adsorption.^2^ The CO_2_ can be selectively trapped using methods such as adsorption, cryogenic capture, and membrane separation in DAC or from gas mixtures. Significant research is being conducted utilizing a diverse array of materials for the capture of carbon dioxide, such as metal–organic frameworks (MOFs), porous silica based (SBA-16, SBA-15 (SBA-Santa Barbara Amorphous)),^3^ MCM-41^4^ (MCM-Mobil Composition of Matter), zeolites, etc. Alkanolamine solutions for carbon dioxide capture represent a sophisticated advancement in this domain.^5^ Nonetheless, the challenges associated with high regeneration costs and material degradation during recycling of the adsorbent remain significant barriers to widespread adoption.

Adsorbents that use acid–base interaction are a promising way to capture CO_2,_ and need active sites on the adsorbent that are rich in nitrogen (act as a base), and CO_2_ behaves as a Lewis acid. One promising approach for capturing CO_2_ involves immobilizing amine-containing materials within a high surface area, such as inert porous substances. Immobilizing amine sites address amine leaching and degradation commonly associated with using amine solutions for CO_2_ capture. It offers effective exposure of active sites (N-sites) for CO_2_ adsorption, even at low concentrations of CO_2_, making it a promising avenue for future advancements.^5^ Although numerous materials with amine functionalization have been reported, amine-impregnated porous silica-based adsorbents are gaining attention due to their abundance, high surface area, tunable pore size, and easily engineered morphology. In addition, the surface silanol group in silica allows the functionalization of material for CO_2_ adsorption.

Based on the nature of the interactions between amine source and support material, sorbents can be classified into three distinct categories. Class-1 sorbents are characterized by a physical interaction between the amine-containing material and the solid support.^4^ Class-2 sorbents involve the formation of covalent bonds between aminosilanes and hydroxyl groups present on the solid support.^5^ The physical impregnation of amines (class-1) and grafting amines (class-2) on porous silica has been widely reported for CO_2_ capture. Class-3 materials are produced via the in situ polymerization of amine monomers on a porous support. Recently, a novel hybrid classification has been introduced, which combines elements of both class-1 and class-2 sorbents. The new category, termed class-4, exhibits both physical interactions and covalent tethering. The common amine sources used for the CO_2_ capture are diethanolamine (DEA), triethanolamine (TEA), diethylenetriamine (DETA), pentaethylenehexamine (PEHA), tetraethylenepentamine (TEPA), and polyethylenimine (PEI).

### 1.1 From Conventional Spectroscopy to Advanced Solid-State NMR Spectroscopy

Scaroni and co-workers were among the pioneers who used PEI-impregnated silica sorbent for CO_2_ capture.^4^ They used MCM-41 treated with PEI to capture CO_2_. To check the material’s properties, they employed powder X-ray diffraction (PXRD), N_2_ adsorption/desorption, and thermogravimetric analysis (TGA). The PXRD pattern of MCM-41 remained largely unchanged following the incorporation of polyethylenimine (PEI), with only a minor shift in the diffraction peak to a higher angle and a slight decrease in the intensity of peaks. These observations suggest that the mesoporous structure of MCM-41 retains its stability upon PEI loading, while the shift and intensity reduction indicate successful filling of the pores with PEI. The above-mentioned stability highlights the structural integrity of the material and its suitability for applications requiring pore modification without compromising the framework. The N_2_ adsorption/desorption studies showed a reduction in pore volume and size, confirming that PEI occupies the pores.

The characterization of polymer impregnation within porous material is commonly conducted using Brunaur-Emmett-Teller (BET) and powder X-ray diffraction (PXRD) measurements. However, PXRD does not reveal if the pores are blocked by the polymer or any other foreign body or if the polymer is properly interacting with the pore walls. Meanwhile, BET measurements provide details about surface area, pore size, and volume but do not confirm any chemical interactions between the polymer and the pore walls. In summary, PXRD and BET measurements give insight into the physical properties of both impregnated and nonimpregnated materials. However, they lack information about chemical bonds and specific proof of proper impregnation and bonding happening inside the pore.

An infrared study of PEI-loaded SBA-15 could provide insights into the chemical interactions occurring between the surface silanol groups and the amine sites in PEI. The absence of infrared (IR) spectroscopy signals from isolated silanol groups postimpregnation and the emergence of new bands corresponding to protonated amine groups are strong indicators of the chemical interaction between the amine and the silica surface. Infrared spectroscopy serves as an effective technique for investigating the interaction between amines and silica surfaces.

**Figure 1:**
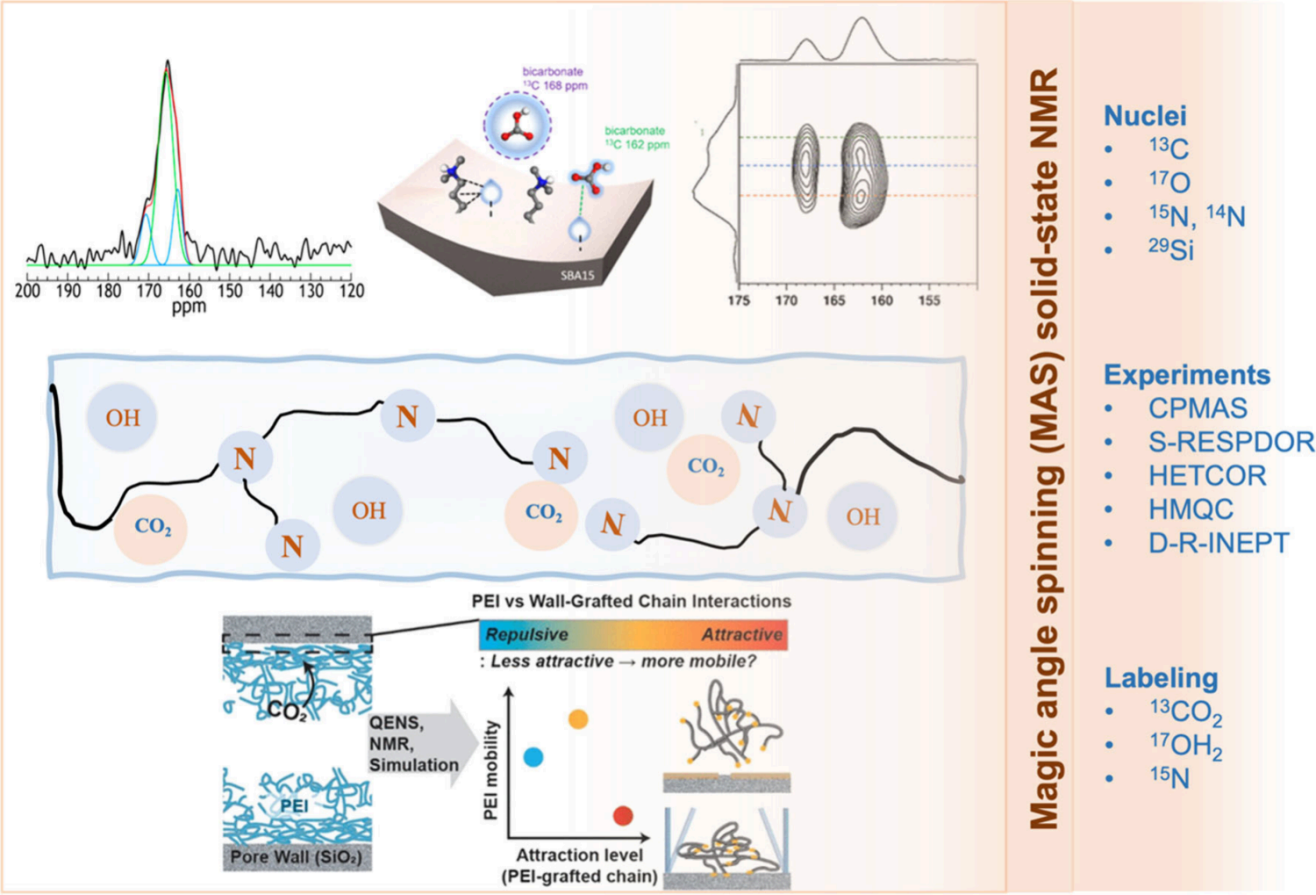
Illustration of various solid-state NMR techniques employed to understand the structural and functional properties of amine–silica-based CO_2_ adsorbents. Adapted with permission from refs (13, 14, and 22). Copyright 2015, 2021, and 2022 American Chemical Society.

Solid-state NMR is an effective method to investigate molecular interactions, mechanisms and dynamic processes in materials. The presence of NMR-active nuclei like ^1^H, ^13^C and ^14^N allows for detailed exploration of these systems through multiple experimental techniques. Interactions between amine and silica could be explored by ^1^H, ^13^C CP (cross-polarization) MAS, ^1^H–^1^H double quantum-single quantum, ^1^H–^29^Si, and ^1^H–^13^C correlation NMR measurements. The mechanism of CO_2_ adsorption can be understood by studying the formation of carbamate and bicarbonate species. In principle, the primary, secondary, or tertiary amines involved in CO_2_ capture can be distinguished using ^1^H NMR, allowing differentiation between physisorption and chemisorption processes. In addition to the experiments mentioned above, it is possible to use ^1^H{^14^N} D-HMQC, D-R-INEPT, and ^1^H{^14^N} RESPDOR to understand the structural changes happening around the ^14^N quadrupolar nuclei. ^1^H{^14^N} D-HMQC, D-R-INEPT, and ^1^H{^14^N} RESPDOR are different advanced solid-state NMR correlation experiments that can spatially link different nuclei in the material. A summary of the various NMR experiments used to characterize the amine–silica-based CO_2_ adsorbents is shown in Figure 1.

Solid-state NMR is a valuable technique for investigating the correlation between proximate proton pairs and examining long-range correlations. Brown and colleagues have demonstrated that a fast MAS experiment at a frequency of 70 kHz can effectively probe the proximities of proton–proton and nitrogen-proton interactions within molecular solids.^6^ They proposed two experimental methodologies. The first is a two-dimensional experiment that aims to correlate the protons on the nitrogen using a ^14^N HMQC (heteronuclear multiple-quantum coherence) filtered ^1^H Double-Quantum (DQ) experiment. The second methodology is a three-dimensional experiment designed to correlate the chemical shifts of ^1^H–^1^H single-quantum (SQ)-double-quantum (DQ) with those of ^14^N.

Investigating the correlation between nitrogen and proton nuclei in amine-based silica sorbents is crucial. Recent advancements in fast MAS NMR experiments allow for the study of ^14^N and ^1^H correlations in these systems. Nishiyama and co-workers studied pharmaceutical compounds using fast MAS NMR experiments.^7^ In this study, they employed a PM-S-RESPDOR (pulse modulated-symmetry based-rotational-echo saturation-pulse double-resonance) to excite the protons near ^14^N. Subsequently, proton magnetization was transferred to adjacent proton sites through the application of a radio frequency-driven recoupling (RFDR) sequence. Furthermore, a combination of the PM-S-RESPDOR and RFDR sequences, in conjunction with a Back-to-Back (BaBa) sequence, was utilized to investigate the local structure and to elucidate the ^1^H–^1^H proximities. Recent advancements in fast MAS NMR techniques for studying intermolecular interactions are highly promising. These experiments combine the benefits of HMQC and proton-based spin diffusion sequences.^8^ This methodology enables the acquisition of both 2D ^1^H{X}-HMQC filtered ^1^H–^1^H correlation spectra and 2D ^1^H{X} HMQC spectra.

## 2 Silica Support Analysis

The characterization of coordination environments and the structural properties of silicon within the silica support can be effectively conducted using ^29^Si MAS NMR spectroscopy. The technique of ^29^Si MAS NMR serves as an effective approach for investigating the various electronic and geometric silicon environments by analyzing the chemical shift of Si. ^29^Si MAS NMR can be utilized to characterize the silicon environment and the changes associated with incorporating elements like aluminum and alkalis into calcium-alumino-silicates. The coordination environment of silica is commonly denoted by the notation “Q^*n*^” and the “*n*” represents the number of oxygen atoms that are bridged to other silicon atoms. Consequently, Q^0^ represents isolated SiO_4_^4–^ unit, while Q^4^ indicates a highly three-dimensional silicate structure. Kennedy and co-workers investigated the dependence of silicon coordination on the extent of dissolution of magnesium silicates by primarily focusing on ^29^Si MAS NMR.^9^ The chemical shift of silicon is highly sensitive to its coordination environment, allowing for the differentiation among the Q^0^ to Q^4^ silica environments. By tracking the transformation of the chemical shifts associated with Q^*n*^ (where *n* = 0, 1, 2, 3, 4) using ^29^Si MAS NMR spectroscopy, it is possible to effectively track the phase transitions and changes in coordination within silica. Laassiri and co-workers presented detailed insights regarding the Q^2^, Q^3^, and Q^4^ silicon species, as revealed by the ^29^Si MAS NMR spectrum of the SBA-15 sample.^10^ They tracked the changes in the local Si environment during the ammonolysis using ^29^Si and ^1^H MAS NMR.

^29^Si MAS NMR was performed on mesoporous SBA-15 to investigate the modifications in the chemical nature of functionalized silica prior to and subsequent to water intrusion.^11^ The resonances associated with the Q^3^ and Q^4^ groups of condensed silica, along with their integrals, remained consistent (within the error limit) before and after the water intrusion. This observation confirms that the chemical nature of the functionalized silica remained unchanged following treatment. The application of ^29^Si MAS NMR is highly effective in examining the changes in the local structure of silicon within silica support.

## 3 Solid-State NMR to Study the Local Structures of CO_2_ Adsorption Species Formed

**Figure 2:**
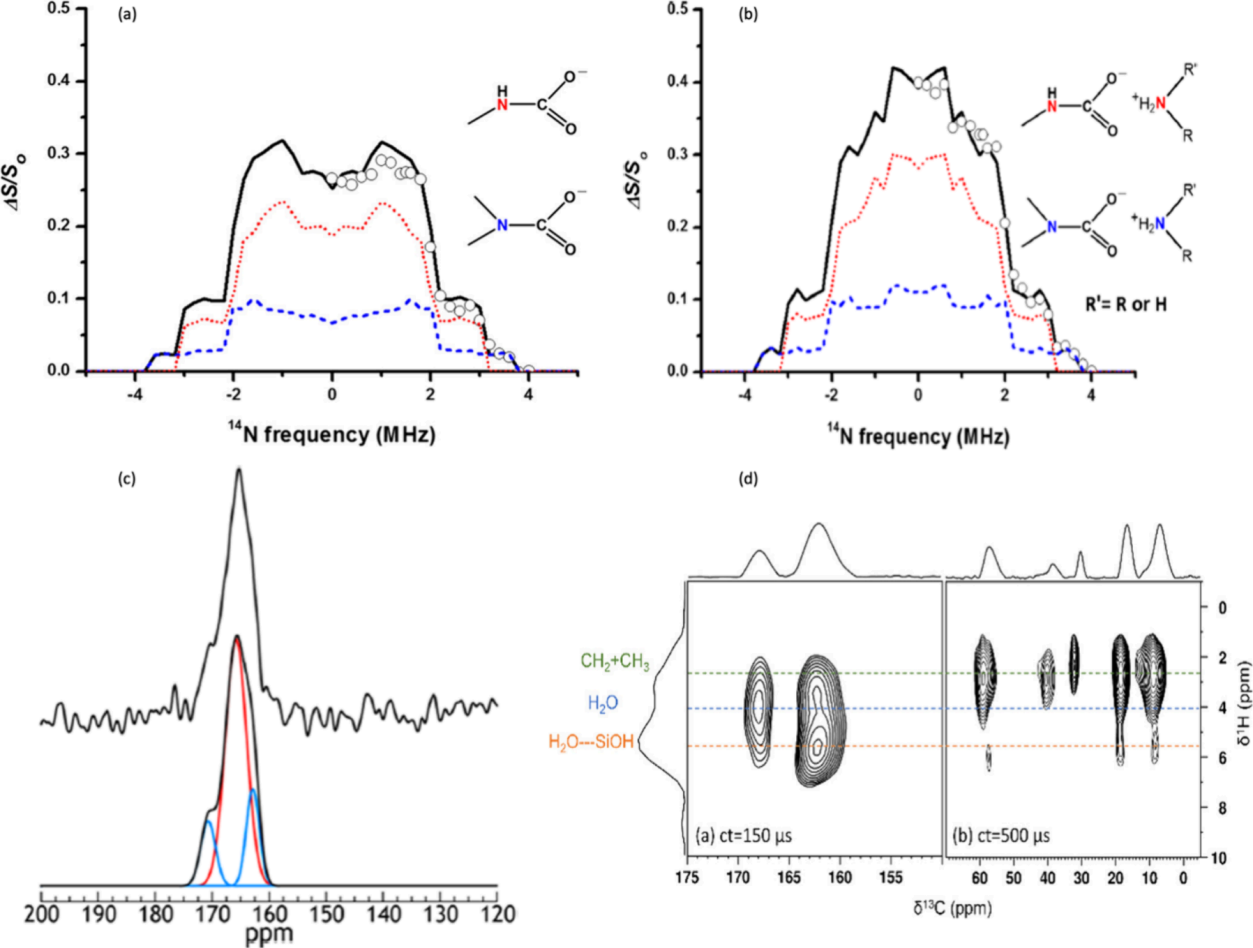
Various solid-state NMR experiments used to investigate the CO_2_ adsorption species on amine–silica material. The ^13^C{^14^N} wide-line NMR spectrum of ^13^CO_2_ adsorbed on TEPA-SBA-15 recorded by the S-RESPDOR NMR sequence with a dephasing time of (a) τ = 3.20 ms and (b) τ = 11.52 ms. The dotted and dashed curves are simulated spectra for secondary, tertiary carbamate-ammonium ion pairs. Adapted with permission from ref (12). Copyright 2014 American Chemical Society. (c) ^1^H-^13^C CPMAS NMR (3 T) of the reacted SBA-15-HAS after evacuating the reacted sample for 31 h. Adapted with permission from ref (13). Copyright 2015 American Chemical Society. (d) The ^1^H-^13^C HETCOR solid-state NMR spectra recorded at 100 K with different contact times of 150 (left) and 500 μs (right). Adapted with permission from ref (14). Copyright 2021 American Chemical Society.

An understanding of the local structure of CO_2_-adsorbed species on amine–silica sorbents is crucial for the design of adsorbents with desired control on CO_2_ adsorption. The CO_2_ adsorption species on amines varies with the presence of moisture; with H_2_O, bicarbonate forms, while in its absence, carbamate or urea is formed. The ^13^C–^14^N symmetry-based rotational-echo saturation-pulse double-resonance (S-RESPDOR) experiment provides valuable information on the different nitrogen environments and, hence, the various types of adsorption species. The S-RESPDOR experiment can be used to measure the dipolar coupling between different nuclei. Therefore, the S-RESPDOR and DFT calculations were utilized to study the CO_2_ adsorption species on mesoporous silica SBA-15 impregnated with tetraethylenepentamine (TEPA).^12^ The study systematically evaluated the spatial proximities between ^13^C and ^14^N nuclei and characterized the quadrupolar parameters of ^14^N to elucidate the mechanisms of CO_2_ adsorption on amine-functionalized silica materials. The spectra were recorded using two distinct dephasing times of 3.20 and 11.52 ms, represented in Figure 2(a),(b). The features at 3.10 and 3.70 MHz in Figure 2(a) suggest the presence of two nitrogen sites. Spectral simulations confirm the presence of two distinct CO_2_ adsorption sites forming carbamates, each characterized by unique quadrupolar coupling constants. The 11.52 ms dephasing time (Figure 2(b)) shows carbon coupling with a distant nitrogen site. In conclusion, primary amines are preferred for CO_2_ adsorption, forming stable secondary carbamates (70% domination) compared to tertiary ones. Optimizing CO_2_ adsorption performance can be achieved by adjusting primary and secondary amine groups. Direct evidence for distinct nitrogen environments can be attained through ^1^H{^14^N} D-HMQC or T-HMQC experiments.

On the other hand, Hayes and co-workers employed ^13^C MAS NMR to explore the adsorption species in Hyperbranched Amine Polymer (HAS)-SBA-15 sorbents (Figure 2(c)).^13^ They recognized several chemisorbed species, including carbamate and carbamic acid, as well as another resonance likely associated with an additional carbamate or bicarbonate. At lower magnetic fields, the splitting of the carbamate ^13^C signal resulting from ^13^C–^14^N dipolar coupling assisted in differentiating the various adsorption products.

Investigating how to provide spectroscopic evidence to distinguish between carbamate and bicarbonate, as well as various bicarbonate and carbamate environments in different amine-based silica sorbents during CO_2_ adsorption, is still challenging. Further, two-dimensional ^1^H–^13^C HETCOR NMR experiments were conducted at low temperatures to examine bicarbonate environments in primary, secondary, and tertiary amines with excess water, using labeled ^13^CO_2_ and D_2_O.^14^ Solid-state NMR experiment has identified different bicarbonates in the pores and on the surface of SBA-15. By varying contact time during the ^1^H–^13^C HETCOR experiment, it is possible to probe both nearby and distant correlations between proton and carbon. Figure 2(d) illustrates this, with the left spectrum representing short contact time and the right showing longer contact time. The ^13^C chemical shift at 162 and 168 ppm indicate two distinct bicarbonate environments. The experimental setup used in the study indicates that it was a model system and does not perfectly replicate the actual CO_2_ adsorption experiments. Mafra and co-workers studied the CO_2_ adsorption species formed by providing a controlled partial pressure of CO_2_ and H_2_O on the CO_2_ adducts formed in amine–silica (SBA-15/APTES, 3-DEAPTES) sorbents.^15^ It helped to understand how moisture-induced bicarbonate species are formed and stabilized on the amine-modified support. The research delivered direct chemical shift evidence that under humid conditions, primary amines combine with carbon dioxide (CO_2_) to create alkylammonium carbamate ion pairs. However, tertiary amines form bicarbonate and a carbamic acid-like species. Evidence for species formation beyond bicarbonate in the presence of water comes from unique experimental methods and NMR spectrum analysis combined with modeling. The ^1^H–^13^C HETCOR experiment represents a powerful technique for investigating carbon–proton correlations. By systematically varying the contact time in ^1^H–^13^C HETCOR experiments enables selective probing of ^1^H–^13^C interactions at varying proximities, thereby distinguishing between nearby and distant nuclear pairs. Consequently, a meticulous analysis of the resulting spectra can elucidate the structural characteristics of adsorbed species, monitor structural changes throughout adsorption–desorption cycles, and assess degradation phenomena at elevated temperatures, among other applications.

## 4 Solid-State NMR Investigation of Amine Immobilization and Stabilization on Silica Sorbents

**Figure 3:**
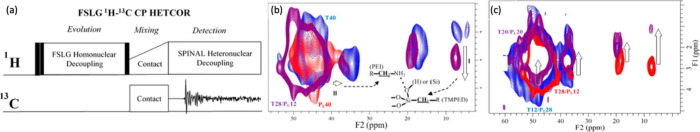
FSLG ^1^H–^13^C CP HETCOR solid-state NMR spectra for the study of the stability of sorbents. (a) The pulse sequence for collecting FSLG ^1^H–^13^C CP HETCOR NMR spectra. (b) Solid state two-dimensional FSLG ^1^H–^13^C CP HETCOR NMR spectra of P_L_40, T40, and T28/P_L_12 sorbents. (c) Solid state 2-dimensional FSLG ^1^H–^13^C CP HETCOR NMR spectra of hybrid sorbents with different TMPED/PEI weight ratios. F1 and F2 indicate the ^1^H and ^13^C axes, and hollow arrows represent cross-peak shifts. Adapted with permission from ref (16). Copyright 2016 American Chemical Society.

Hybrid class-1/class-2 materials (also called class-4) has shown higher stability toward CO_2_ adsorption. However, the amine immobilization and stabilization mechanism of these materials can be studied using solid-state NMR. Gray and co-workers used ^29^Si CP-MAS and two-dimensional FSLG ^1^H–^13^C CP HETCOR NMR measurements to investigate the class-4 materials (Figure 3(a)).^16^ The FSLG ^1^H–^13^C CP HETCOR experiment provides information about the long-range intramolecular interaction between TMPED (N-(3-(trimethoxysilyl)propyl)ethylenediamine) and PEI by probing the alkyl group environments (Figure 3(b)). The P_L_40 notation is used only for class-1 (PEI), while T40 represents materials that are exclusively class-2 (TMPED). Hybrid materials combining class-1 and class-2 are indicated by a mixture of T and P_L_. The observed shift in the peak at the alkyl position, both prior to and subsequent to the formation of the hybrid material (T28/P_L_12), serves as compelling evidence for the formation of PEI–NH_2_···NH_2_–TMPED species, as well as the direct proof of the PEI–NH_2_···HO–Si/O–Si–O linkages. Figure 3(c) is the comparison of hybrid class-1/class-2 materials with different ratios of TMPED and PEI. The hydrogen-bonded PEI–NH_2_···NH_2_–TMPED and PEI–NH_2_···HO–Si/O–Si–O linkages within the hybrid class-1/class-2 sorbent are the reason for their higher stability.

## 5 Identification of Different Protonated States of CO_2_ on Adsorbent and Adsorption Isotherm

**Figure 4:**
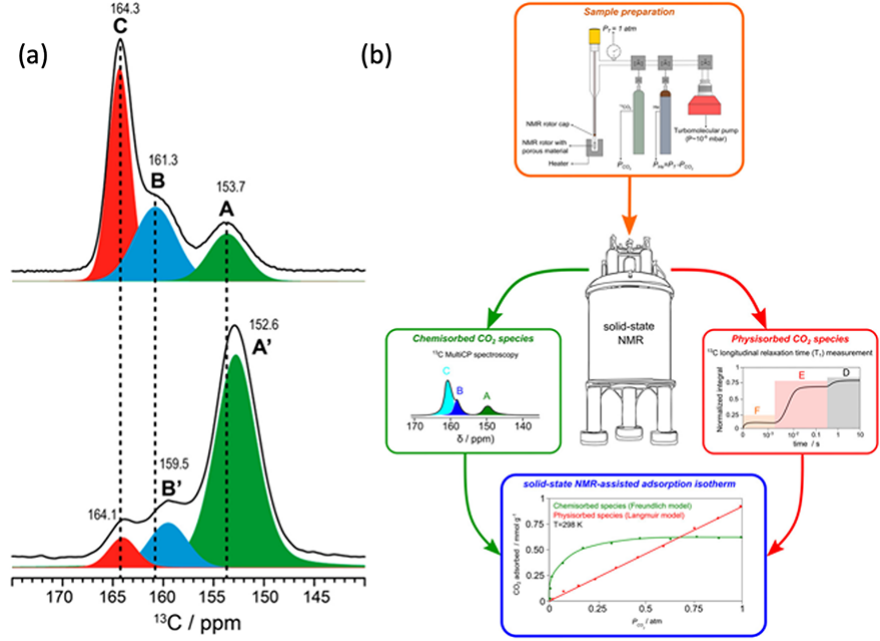
(a) The ^13^C CPMAS NMR spectra of ^13^CO_2_-loaded (3-aminopropyltriethoxysilane) APTES@SBA-15 with high (top) and low (bottom) amine loadings. Adapted with permission from ref (18). Copyright 2019 American Chemical Society. (b) Schematic representation of the methodology used for the qualitative and quantitative characterization of chemisorbed and physisorbed CO_2_ species formed in different partial pressures of CO_2_. Adapted with permission from ref (19). Copyright 2023 American Chemical Society.

NMR spectroscopy can be used to differentiate between various protonated states of CO_2_ on amine–silica surfaces. The utilization of CSA tensors for proton transfer to distinguish between ionic/charged and neutral CO_2_ species, which are formed upon the adsorption of ^13^CO_2_ in amine-modified porous materials, has been successfully demonstrated by Mafra and co-workers.^17^ The first evidence for isolated chemisorbed CO_2_ species was achieved by using bulky amine protection groups in the synthesis. The ^13^C species appearing at 164 ppm is the ammonium carbamate ion pair ([R–NH–COO][NH_3_R]), ^13^C species appearing at 160 ppm is a hydrogen-bonded species (−R–NH–COOH···NH_2_R), and species appearing at 154 ppm predominantly due to the interaction between carbamic acid and silanol (−R–NH–COOH). The ^13^C CSA tensor elements distinguish these protonation species on the amine–silica sorbent surface. After carefully packing solid-state NMR rotor with no moisture and controlled CO_2_ partial pressures, an experiment could record a ^13^C CP spectrum, as shown in Figure 4(a).^18^ Materials with higher amine loadings, species A and B, are identified as carbamic acid. As the amine concentration decreases, their associated peaks shift to lower chemical shift values, while ammonium carbamate species remain unchanged. The intensity difference in amine peaks 164 ppm at two different amine loadings indicates the disappearance of paired amines with lower concentrations. They provided experimental evidence for previously reported adsorption species. The ability to differentiate between chemisorbed and physisorbed CO_2_ using NMR spectroscopy is unique. Recently, solid-state NMR was used to generate adsorption isotherms for CO_2_ adsorption on amine–silica sorbents for the first time.^19^ A schematic representation of the methodology used is provided in Figure 4(b). This research underscores the distinct capability of solid-state NMR to differentiate between individual isotherms associated with chemisorbed and physisorbed CO_2_, a distinction that can be achieved through the utilization of NMR techniques. The quantification of chemisorbed species through cross-polarization experiments and physisorbed species using *T*_1_ measurement is utilized to plot isotherms.NMR spectroscopy can differentiate between various protonated states of CO_2_ on amine–silica surfaces by analyzing the chemical shift anisotropy (CSA) tensor

## 6 Insights from Chemical Shift Anisotropy and Relaxation Time

**Figure 5:**
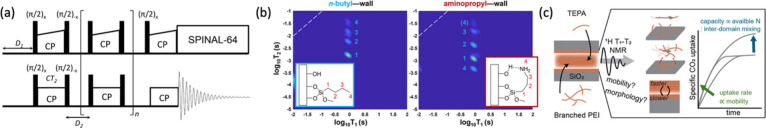
(a) Schematic for multiple cross-polarization pulse sequences used to get ^13^C MAS NMR spectra. Adapted with permission from ref (20). Copyright 2021 American Chemical Society. (b) *T*_1_*–T*_2_ relaxation correlation plots corresponding to the different molecules grafted on the SBA-15 wall. Left, SBA-15-CH_3_ (*n*-butyl) and right SBA-15-NH_2_ (aminopropyl), where signal “4” represents proton of amine groups interacting with SBA-15 walls while signal “(4)” represents amino protons, not actively interacting with walls. Adapted with permission from ref (22). Copyright 2022 American Chemical Society. (c) Probing the Morphology and Mobility of Amines in Porous Silica CO_2_ Sorbents by ^1^H *T*_1_*–T*_2_ Relaxation Correlation NMR, an overall depiction. Adapted with permission from ref (23). Copyright 2023 American Chemical Society.

Solid-state NMR offers insights into weakly interacting CO_2_ on amine–silica sorbents by utilizing chemical shift anisotropy (CSA) and longitudinal relaxation times (*T*_1_).^20^ The multi-CP enhancement method developed by Johnson and Schmidt-Rohr was used in CO_2_ adsorption studies for the first time.^21^ The unique pulse sequence utilized in multiple cross-polarization NMR experiments is illustrated in Figure 5(a). The research marks the first instance of quantifying and identifying various physisorbed species through CSA and *T*_1_ measurements. Poly(ethylenimine) (PEI) impregnated silica sorbents are notable materials for CO_2_ adsorption. Understanding PEI’s interaction with its support is crucial for optimizing its adsorption efficiency. This can be studied using ^1^H *T*_1_*–T*_2_ relaxation correlation solid-state NMR and other spectroscopic methods. Jones and co-workers utilized quasi-elastic neutron scattering (QENS), solid-state NMR, and molecular dynamics (MD) simulations to examine PEI’s behavior within the pores.^22^Figure 5(b) is the *T*_1_*-T*_2_ plots for different chains grafted on silica support. The left plot in Figure 5(b) belongs to *n*-butyl grafted, and the right one is of aminopropyl grafted silica wall. The *T*_1_*-T*_2_ measurement is used to study the interaction of amine with the hydrophilic walls of SBA-15. The *T*_2_ of the amino proton, which is not actively interacting with the surface of the support material, is more mobile and is different (‘(4)’) from that of the amino protons that are interacting with the wall (‘4’). PEI-silica materials exhibit a rapid CO_2_ uptake followed by a gradual stage, where the initial phase is driven by vacant amine sites and the latter by CO_2_ diffusion limits. The same group recently investigated the mobility and dynamics of amine–silica sorbents using ^1^H *T*_1_*-T*_2_ relaxation correlation solid-state NMR.^23^ The overall depiction of the study is given in Figure 5(c). They concluded that when there is a higher concentration of amine filling the pores, the interaction with the pore wall decreases, or that the amine becomes more mobile due to interdomain mixing effects.

## 7 Conclusions and Future Outlook

Recently, solid-state NMR has emerged as a valuable spectroscopic tool for unveiling the topology of complex molecules.^24,25^ In this mini-review, we explained how various solid-state NMR experiments have been utilized to understand the structure of amine–silica based adsorbents and the CO_2_ adsorption species formed. The nitrogen atom’s chemical environment and the changes it undergoes under different conditions directly influence the adsorption species formed after CO_2_ incorporation. Since the amines serve as the active sites in CO_2_ adsorbents, so investigating the nitrogen environment through solid-state NMR experiments provides insights into the CO_2_ adsorption mechanism. Solid-state NMR techniques such as ^1^H{^14^N} RESPDOR experiments,^26^^1^H{^14^N} D-HMQC and T-HMQC experiments are effective for evaluating nitrogen-proton correlations in various environments.

Exploring ^17^O NMR can be particularly beneficial for detecting various oxygen environments. This is because the oxygen atoms in CO_2_ can experience different environments upon adsorption.^2^ We also recommend using ^17^O labeling on the hydroxyl groups present on the surface of silica. This method creates synthetic difficulties due to the labeling that occurs after the synthesis of the silica adsorbent. The uniform enrichment of ^17^O can be achieved through repeated thermal dehydration and rehydration of a silica sample using ^17^OH_2_.^27^ An alternative approach involves the utilization of ^17^O_2_ gas at elevated temperatures. Most of the methods currently employed are expensive, as they require ^17^OH_2_ or ^17^O_2_ at elevated temperatures. Recently, Perras and co-workers have demonstrated that silanols on the surface of silica can be selectively exchanged with ^17^OH_2_ at room temperature.^28^ This method is much more efficient and cost-effective as it does not require breaking Si–O–Si linkages.

The real-time observation of the adsorption process, particularly through in situ spectroscopic experiments, can provide valuable insights into the formation and behavior of intermediates. However, the challenge lies in adapting solid-state NMR techniques for such in situ experimental setups. Specifically, applying these methods to study CO_2_ adsorption at varying temperatures presents significant engineering hurdles that must be overcome to achieve reliable and informative results.

In the past decade, Dynamic Nuclear Polarization (DNP) has emerged as a method capable of increasing sensitivity by two to 3 orders of magnitude. In DNP, polarization is transferred from an electron spin ensemble to a nuclear spin ensemble under microwave irradiation. The high gyromagnetic ratio of electrons compared to nuclei gives electron spins significantly greater polarization, resulting in a substantial increase in nuclear sensitivity. For the DNP MAS NMR experiments the material of interest should have a paramagnetic agent, which may be naturally occurring within the material (endogenous DNP) or introduced externally through the addition of a polarizing agent (exogenous DNP). Sayari and co-workers have recently reported the electron paramagnetic resonance (EPR) spectra of free radical electrons within the polyethylenimine (PEI)-silica system upon heating.^29^ The generated radicals can be utilized in advanced endogenous DNP experiments. This method can offer deeper insights into the mechanisms of degradation and the structural changes in PEI-silica materials. The application of exogenous surface-enhanced silicon-29 DNP NMR spectroscopy to silica, utilizing TOTAPOL as a polarizing agent can be explored. Additionally, simulating NMR chemical shifts using various models can provide valuable insights into the underlying mechanisms.

Employing high magnetic fields can further enhance these studies by effectively scaling the quadrupolar coupling constants of nuclei such a ^17^O and ^14^N nuclei. Recently, Reddy and co-workers have shown improved spectral resolution when a high magnetic field of 28.2 T is used to characterize the local structures in xerogels.^30^ They developed dual cross-linked cellulose-based hydrogel using carboxymethylcellulose (CMC), where citric acid and Al^3+^ are cross-linkers. In this study, a high magnetic field of 28.2 T was employed to obtain one-dimensional and two-dimensional solid-state NMR spectra for both ^1^H and ^27^Al nuclei. Additionally, a magnetic field of 21.2 T was utilized for the acquisition of spectra from citric acid powder, specifically focusing on one-dimensional ^1^H and two-dimensional ^1^H–^1^H correlations. Furthermore, a magnetic field of 18.8 T was used to investigate the ion exchange properties of the materials studied. The application of a high magnetic field has effectively resolved the overlapping chemical shifts in complex materials, such as dual-cross-linked hydrogels.

In conclusion, solid-state NMR represents an essential spectroscopic technique for the investigation of complex systems, such as amine–silica adsorbents. Advancements in solid-state NMR spectroscopy could significantly enhance our understanding of carbon dioxide adsorption mechanisms on amine–silica materials.

## References

[ref1] Langie K. M. G.; Tak K.; Kim C.; Lee H. W.; Park K.; Kim D.; Jung W.; Lee C. W.; Oh H.-S.; Lee D. K.; et al. Toward economical application of carbon capture and utilization technology with near-zero carbon emission. Nat. Commun. 2022, 13 (1), 7482 10.1038/s41467-022-35239-9. 36470930 PMC9722933

[ref2] Berge A. H.; Pugh S. M.; Short M. I.; Kaur C.; Lu Z.; Lee J.-H.; Pickard C. J.; Sayari A.; Forse A. C. Revealing carbon capture chemistry with 17-oxygen NMR spectroscopy. Nat. Commun. 2022, 13 (1), 7763 10.1038/s41467-022-35254-w. 36522319 PMC9755136

[ref3] Jahandar Lashaki M.; Sayari A. CO_2_ capture using triamine-grafted SBA-15: The impact of the support pore structure. Chem. Eng. J. 2018, 334, 1260–1269. 10.1016/j.cej.2017.10.103.

[ref4] Xu X.; Song C.; Andresen J. M.; Miller B. G.; Scaroni A. W. Novel polyethylenimine-modified mesoporous molecular sieve of MCM-41 type as high-capacity adsorbent for CO_2_ capture. Energy Fuels 2002, 16 (6), 1463–1469. 10.1021/ef020058u.

[ref5] Cherevotan A.; Raj J.; Peter S. C. An overview of porous silica immobilized amines for direct air CO_2_ capture. J. Mater. Chem. A 2021, 9 (48), 27271–27303. 10.1039/D1TA05961K.

[ref6] Reddy G. M.; Malon M.; Marsh A.; Nishiyama Y.; Brown S. P. Fast Magic-Angle Spinning Three-Dimensional NMR Experiment for Simultaneously Probing H-H and N-H Proximities in Solids. Anal. Chem. 2016, 88 (23), 11412–11419. 10.1021/acs.analchem.6b01869. 27797191

[ref7] Hong Y.-l.; Reddy G. M.; Nishiyama Y. Selective detection of active pharmaceutical ingredients in tablet formulations using solid-state NMR spectroscopy. Solid State Nucl. Magn. Reson. 2020, 106, 101651 10.1016/j.ssnmr.2020.101651. 32058901

[ref8] Raval P.; Trébosc J.; Pawlak T.; Nishiyama Y.; Brown S. P.; Reddy G. M. Combining heteronuclear correlation NMR with spin-diffusion to detect relayed Cl–H–H and N–H–H proximities in molecular solids. Solid State Nucl. Magn. Reson. 2022, 120, 101808 10.1016/j.ssnmr.2022.101808. 35780556

[ref9] Benhelal E.; Hook J. M.; Rashid M. I.; Zhao G.; Oliver T.; Rayson M.; Brent G.; Stockenhuber M.; Kennedy E. Insights into chemical stability of Mg-silicates and silica in aqueous systems using ^25^Mg and ^29^Si solid-state MAS NMR spectroscopy: Applications for CO_2_ capture and utilisation. Chem. Eng. J. 2021, 420, 127656 10.1016/j.cej.2020.127656.

[ref10] Sfeir A.; Teles C. A.; Ciotonea C.; Reddy G. M.; Marinova M.; Dhainaut J.; Löfberg A.; Dacquin J.-P.; Royer S.; Laassiri S. Enhancing ammonia catalytic production over spatially confined cobalt molybdenum nitride nanoparticles in SBA-15. Appl Catal., B 2023, 325, 122319 10.1016/j.apcatb.2022.122319.

[ref11] Collados C. C.; Huber C.; Söllner J.; Grass J.-P.; Inayat A.; Durdyyev R.; Smith A.-S.; Wisser D.; Hartmann M.; Thommes M. Assessment of Hydrophilicity/Hydrophobicity in Mesoporous Silica by Combining Adsorption, Liquid Intrusion, and Solid-State NMR Spectroscopy. Langmuir 2024, 40, 12853 10.1021/acs.langmuir.3c03516. 38861921

[ref12] Huang S.-J.; Hung C.-T.; Zheng A.; Lin J.-S.; Yang C.-F.; Chang Y.-C.; Deng F.; Liu S.-B. Capturing the local adsorption structures of carbon dioxide in polyamine-impregnated mesoporous silica adsorbents. J. Phys. Chem. Lett. 2014, 5 (18), 3183–3187. 10.1021/jz501616c. 26276330

[ref13] Moore J. K.; Sakwa-Novak M. A.; Chaikittisilp W.; Mehta A. K.; Conradi M. S.; Jones C. W.; Hayes S. E. Characterization of a mixture of CO_2_ adsorption products in hyperbranched aminosilica adsorbents by ^13^C solid-state NMR. Environ. Sci. Technol. 2015, 49 (22), 13684–13691. 10.1021/acs.est.5b02930. 26477882

[ref14] Chen C.-H.; Sesti E. L.; Lee J. J.; Mentink-Vigier F.; Sievers C.; Jones C. W.; Hayes S. E. NMR reveals two bicarbonate environments in SBA15-solid-amine CO_2_ sorbents. J. Phys. Chem. C 2021, 125 (30), 16759–16765. 10.1021/acs.jpcc.1c04145.

[ref15] Sardo M.; Afonso R.; Juźków J.; Pacheco M.; Bordonhos M.; Pinto M. L.; Gomes J. R.; Mafra L. Unravelling moisture-induced CO_2_ chemisorption mechanisms in amine-modified sorbents at the molecular scale. J. Mater. Chem. A 2021, 9 (9), 5542–5555. 10.1039/D0TA09808F. PMC8459418 34671479

[ref16] Wilfong W. C.; Kail B. W.; Jones C. W.; Pacheco C.; Gray M. L. Spectroscopic investigation of the mechanisms responsible for the superior stability of hybrid class 1/class 2 CO_2_ sorbents: a new class 4 category. ACS Appl. Mater. Interfaces 2016, 8 (20), 12780–12791. 10.1021/acsami.6b02062. 27145200

[ref17] Čendak T.; Sequeira L.; Sardo M.; Valente A.; Pinto M. L.; Mafra L. Detecting Proton Transfer in CO_2_ Species Chemisorbed on Amine-Modified Mesoporous Silicas by Using ^13^C NMR Chemical Shift Anisotropy and Smart Control of Amine Surface Density. Chem. - Eur. J. 2018, 24 (40), 10136–10145. 10.1002/chem.201800930. 29663545

[ref18] Afonso R.; Sardo M.; Mafra L.; Gomes J. R. Unravelling the structure of chemisorbed CO_2_ species in mesoporous aminosilicas: a critical survey. Environ. Sci. Technol. 2019, 53 (5), 2758–2767. 10.1021/acs.est.8b05978. 30730709

[ref19] Ilkaeva M.; Vieira R.; Pereira J. M.; Sardo M.; Marin-Montesinos I.; Mafra L. Assessing CO_2_ Capture in Porous Sorbents via Solid-State NMR-Assisted Adsorption Techniques. J. Am. Chem. Soc. 2023, 145 (16), 8764–8769. 10.1021/jacs.3c00281. 37037457 PMC10141401

[ref20] Vieira R.; Marin-Montesinos I.; Pereira J.; Fonseca R.; Ilkaeva M.; Sardo M.; Mafra L. Hidden” CO_2_ in Amine-Modified Porous Silicas Enables Full Quantitative NMR Identification of Physi-and Chemisorbed CO_2_ Species. J. Phys. Chem. C 2021, 125 (27), 14797–14806. 10.1021/acs.jpcc.1c02871. PMC8456409 34567337

[ref21] Johnson R. L.; Schmidt-Rohr K. Quantitative solid-state ^13^C NMR with signal enhancement by multiple cross polarization. J. Magn. Reson. 2014, 239, 44–49. 10.1016/j.jmr.2013.11.009. 24374751

[ref22] Moon H. J.; Carrillo J.-M.; Leisen J.; Sumpter B. G.; Osti N. C.; Tyagi M.; Jones C. W. Understanding the impacts of support–polymer interactions on the dynamics of poly (ethyleneimine) confined in Mesoporous SBA-15. J. Am. Chem. Soc. 2022, 144 (26), 11664–11675. 10.1021/jacs.2c03028. 35729771

[ref23] Moon H. J.; Sekiya R.-S.; Jones C. W. Probing the Morphology and Mobility of Amines in Porous Silica CO_2_ Sorbents by ^1^H *T*_*1*_*–T*_*2*_ Relaxation Correlation NMR. J. Phys. Chem. C 2023, 127 (24), 11652–11665. 10.1021/acs.jpcc.3c02441.

[ref24] Mathew R.; Mazumder A.; Kumar P.; Matula J.; Mohamed S.; Brazda P.; Hariharan M.; Thomas B. Unveiling the topology of partially disordered micro-crystalline nitro-perylenediimide with X-aggregate stacking: an integrated approach. Chem. Sci. 2024, 15 (2), 490–499. 10.1039/D3SC05514K. 38179523 PMC10762722

[ref25] Vinod K.; Mathew R.; Jandl C.; Thomas B.; Hariharan M. Electron diffraction and solid-state NMR reveal the structure and exciton coupling in a eumelanin precursor. Chem. Sci. 2024, 15 (39), 16015–16024. 10.1039/D4SC05453A. 39345764 PMC11423530

[ref26] Gan Z. Measuring multiple carbon–nitrogen distances in natural abundant solids using R-RESPDOR NMR. Chem. Commun. 2006, (45), 4712–4714. 10.1039/B611447D. 17109045

[ref27] Merle N.; Trebosc J.; Baudouin A.; Rosal I. D.; Maron L.; Szeto K.; Genelot M.; Mortreux A.; Taoufik M.; Delevoye L.; et al. ^17^O NMR gives unprecedented insights into the structure of supported catalysts and their interaction with the silica carrier. J. Am. Chem. Soc. 2012, 134 (22), 9263–9275. 10.1021/ja301085m. 22571376

[ref28] Agarwal A.; Mais M.; Perras F. A. Selective ^17^O-labeling of silica. Chem. Commun. 2024, 60 (84), 12189–12192. 10.1039/D4CC04584J. 39344939

[ref29] Yan C.; Sayari A. Spectroscopic investigation into the oxidation of polyethylenimine for CO_2_ capture: Mitigation strategies and mechanism. Chem. Eng. J. 2024, 479, 147498 10.1016/j.cej.2023.147498.

[ref30] Thomas N.; Moussaoui S.; Reyes-Suárez B.; Lafon O.; Reddy G. M. Dual cross-linked cellulose based hydrogel films. Mater. Adv. 2024, 5 (23), 9210–9219. 10.1039/D4MA00815D.

